# Asymmetric wall ingrowth deposition in Arabidopsis phloem parenchyma transfer cells is tightly associated with sieve elements

**DOI:** 10.1093/jxb/erac234

**Published:** 2022-05-24

**Authors:** Xiaoyang Wei, Yuan Huang, Suong T T Nguyen, David A Collings, David W McCurdy

**Affiliations:** Centre for Plant Science, School of Environmental and Life Sciences, The University of Newcastle, Callahan NSW 2308, Australia; College of Horticulture and Forestry Sciences, Huazhong Agricultural University, Wuhan Hubei 430070, China; College of Horticulture and Forestry Sciences, Huazhong Agricultural University, Wuhan Hubei 430070, China; Centre for Plant Science, School of Environmental and Life Sciences, The University of Newcastle, Callahan NSW 2308, Australia; Centre for Plant Science, School of Environmental and Life Sciences, The University of Newcastle, Callahan NSW 2308, Australia; School of Molecular Sciences, The University of Western Australia, Crawley WA 6009, Australia; Harry Butler Institute, Murdoch University, Murdoch WA 6150, Australia; Centre for Plant Science, School of Environmental and Life Sciences, The University of Newcastle, Callahan NSW 2308, Australia; Northwest Agriculture and Forestry University, China

**Keywords:** Arabidopsis, AtSWEET11, AtSUC2, companion cells, minor vein, phloem parenchyma transfer cell, phloem loading, sieve elements, wall ingrowths

## Abstract

In Arabidopsis, polarized deposition of wall ingrowths in phloem parenchyma (PP) transfer cells (TCs) occurs adjacent to cells of the sieve element/companion cell (SE/CC) complex. However, the spatial relationships between these different cell types in minor veins, where phloem loading occurs, are poorly understood. PP TC development and wall ingrowth localization were compared with those of other phloem cells in leaves of Col-0 and the transgenic lines *AtSUC2::AtSTP9-GFP* (green fluorescent protein) and *AtSWEET11::AtSWEET11-GFP* that identify CCs and PP cells, respectively. The development of PP TCs in minor veins, indicated by deposition of wall ingrowths, proceeded basipetally in leaves. However, not all PP cells develop wall ingrowths, and higher levels of deposition occur in abaxial- compared with adaxial-positioned PP TCs. Furthermore, the deposition of wall ingrowths was exclusively initiated on and preferentially covered the PP TC/SE interface, rather than the PP TC/CC interface, and only occurred in PP cells that were adjacent to SEs. Collectively, these results demonstrate a tight association between SEs and wall ingrowth deposition in PP TCs and suggest the existence of two subtypes of PP cells in leaf minor veins. Compared with PP cells, PP TCs showed more abundant accumulation of AtSWEET11–GFP, indicating functional differences in phloem loading between PP and PP TCs.

## Teaser

Wall ingrowth deposition in phloem parenchyma transfer cells of Arabidopsis leaf minor veins is initiated adjacent to sieve elements, rather than companion cells, and expands to preferentially cover this interface.

## Introduction

The phloem is the main long-distance transport system for nutrients in plants. Anatomically, phloem is built up by arrays of companion cells (CCs), sieve elements (SEs), and adherent phloem parenchyma (PP) cells, in which CCs and SEs are connected by plasmodesmata and thus form the SE/CC complex which acts as the fundamental structural and functional unit of the phloem system (van Bel, 2003; Turgeon and Wolf, 2009). In some species, such as pea and *Arabidopsis thaliana* (Arabidopsis), phloem cells, including both CCs and PP cells, deposit extensive networks of wall ingrowths, transdifferentiating into transfer cells (TCs) (Pate and Gunning, 1969; Amiard *et al.*, 2007; Nguyen and McCurdy, 2016). Transdifferentiation is the conversion of one differentiated cell type into another (Nguyen and McCurdy, 2016) and, in Arabidopsis, the transdifferentiation of PP cells into PP TCs occurs exclusively in the minor veins of foliar tissues such as cotyledons and leaves (Haritatos *et al.*, 2000; Nguyen and McCurdy, 2015). A survey of this process revealed that wall ingrowth deposition in these minor veins is a novel trait of heteroblasty, or vegetative phase change whereby young and adult leaves show differences in form (Poethig, 2010), with ingrowths being more abundant in fully mature juvenile leaves compared with mature adult leaves. In addition, the level of wall ingrowth deposition displays a distinct basipetal gradient in mature adult leaves, with ingrowths being more developed in the leaf apices (Nguyen *et al.*, 2017). However, the fine-scale developmental progression of PP TCs during leaf development remains unclear.

TCs undergo wall ingrowth deposition to increase plasma membrane surface area, thus facilitating increased capacity for transmembrane transport. In Arabidopsis, PP TCs in foliar minor veins play important roles in phloem loading by importing sucrose symplasmically from bundle sheath cells then exporting this sucrose into the apoplasm for subsequent uptake by CCs in the SE/CC complex (Chen, 2014). However, wall ingrowth deposition in PP TCs occurs not only along the interface adjacent to CCs but also along the interface adjacent to SEs (Haritatos *et al.*, 2000; Amiard *et al.*, 2007; Nguyen *et al.*, 2017). Given the proposed role of wall ingrowth deposition in phloem loading, their polarized distribution along the interface adjacent to SE in PP TCs is intriguing. Nonetheless, the exact progression of wall ingrowth deposition in the PP TCs, and precise relationships with the abutting SE/CC complex, have not been fully elucidated.

Recent research has demonstrated additional complexity within the organization of vascular bundles within leaves. Chen *et al.* (2018) identified two subtypes of CCs in minor veins of Arabidopsis, those that express *FLOWERING LOCUS T* (*FT*) and those that do not. Similarly, transcriptomes of abaxial and adaxial bundle sheath cells in maize leaf minor veins are distinct from each other (Bezrutczyk *et al.*, 2021). Furthermore, recent single-cell RNA-sequencing (scRNA-Seq) of enriched leaf vascular tissue reported the identification of two clusters of PP cells, one enriched in defence and cell wall genes (PP1 cluster) and one enriched in photosynthesis-related genes (PP2 cluster) (Kim *et al.*, 2021). Consequently, one possibility arising from this study is that not all PP cells in leaf minor veins transdifferentiate to become PP TCs by deposition of wall ingrowths. Since both AtSWEET11 and AtSWEET12 are cell type markers of PP cells in foliar tissues in Arabidopsis (Chen *et al.*, 2012; Cayla *et al.*, 2019), a structural survey of transgenic lines expressing *AtSWEET11-GFP* (green fluorescent protein) or *AtSWEET12-GFP* constructs may answer this question.

In this study, the developmental processes and morphology of PP TCs and their spatial relationship with other phloem cells were surveyed in Arabidopsis leaves. To assess cellular organization in minor veins, vibratome sectioning allowing high spatial resolution of vein structure was undertaken. Additionally, structural studies were conducted on leaf minor veins in transgenic fluorescent plants expressing the *p*AtSUC2::AtSTP9–GFP and *p*AtSWEET11::AtSWEET11–GFP constructs that identify different phloem cell types. Observation and quantification of these lines was possible using ClearSee imaging (Kurihara *et al.*, 2015), which allowed observation of the fluorescent proteins concurrently with stained wall ingrowths. The development of PP TCs was highly asymmetric in both minor veins and leaves, and coincided with the onset of phloem loading activity in the plant. In addition, AtSWEET11 was more abundant in PP TCs compared with PP cells with no wall ingrowths. However, a tight spatial association between wall ingrowth deposition and SEs was observed. These observations indicate that the phloem loading route and phloem organization in Arabidopsis leaf minor veins is complex.

## Materials and methods

### Plant growth and materials

Seed of plants expressing *pAtSUC2::AtSTP9-GFP* (*tmSTP9*) and *pAtSWEET11::AtSWEET11-GFP* were kindly provided by Dr Ruth Stadler (Universität Erlangen-Nürnberg, Germany) and Dr Sylvie Dinant (INRAE, France), respectively, while Col-0, Ws-2, and *suc2-1*^*+/–*^ seeds were supplied by the ABRC. All seeds were sown and germinated in potting mix soil after being stratified in darkness at 4 °C for 48 h. After stratification, all plants were grown in a growth cabinet under standard lighting conditions for Arabidopsis (100–120 μmol m^–2^ s^–1^, 22 °C day/18 °C night, 16 h photoperiod).

### Cross-sectioning and ClearSee treatment

Transition leaves from 4- to 5-week-old seedlings (the precise leaf number and age of seedlings sampled in each experiment are given in the corresponding Results section) of Col-0 and *pAtSUC2::AtSTP9-GFP* were fixed in 4% (w/v) formaldehyde in phosphate-buffered saline (PBS: 137 mM NaCl, 2.7 mM KCl, 10 mM Na_2_HPO_4_, 1.8 mM KH_2_PO_4_) in a vacuum chamber at room temperature for 1 h. Fixed tissues were then washed thoroughly in PBS before being embedded in 4% (w/v) agarose gel blocks prepared in TAE buffer (40 mM Tris-acetate, 1 mM EDTA, pH 8.0). Cross-sections of 150 µm thickness were cut with a Leica VT1200 vibratome.

Mature juvenile leaves 1 and 2 (hereafter described as leaf 1) from 27-day-old *pAtSWEET11::AtSWEET11-GFP* seedlings were sampled for ClearSee treatment. The abaxial epidermis of leaf samples was peeled off as described by Cayla *et al.* (2019). Thereafter, processed samples were fixed with 4% (w/v) formaldehyde in PBS in a vacuum chamber at room temperature for 1 h, washed with PBS for 1 min twice, then cleared in ClearSee solution (0.66 M xylitol, 0.38 M sodium deoxycholate, 4.16 M urea) for at least 4 d as described by Kurihara *et al.* (2015).

### Cell wall labelling and confocal imaging

Propidium iodide (PI) staining of leaf tissues was performed as described by Nguyen and McCurdy (2015). For *pAtSWEET11::AtSWEET11-GFP* plants, cleared samples were stained with 0.05% (w/v) calcofluor white solution for 40 min. After staining, samples were washed in ClearSee solution for 30 min before being mounted on slides in ClearSee for confocal microscopy observation.

Three confocal microscopy systems were used for image collection in this study, namely the Olympus FV1000 and FV3000 systems and a Leica SP8 system. Imaging settings were as follows: PI, excitation: 552 nm, emission window: 570–670 nm; calcofluor white, excitation: 408 nm; emission window: 425–460 nm; GFP, excitation: 488 nm, emission window: 497–527 nm.

### Measurements of cell/cell interface length and fluorescence intensity

Lengths of the PP TC/CC and PP TC/SE interfaces and the relative fluorescence intensity of SWEET11–GFP were measured using the image processing software ImageJ (Fiji version 1.51n).

For the fluorescence intensity assay of SWEET11–GFP, in each comparison set, confocal images from the same sequential scanning were collected for analysis. Confocal images were converted into 8-bit images, and the threshold was adjusted using the default setting of the software. Thereafter, fluorescence intensity of the selected region of interest (ROI) was measured. Adaxially positioned phloem cells imaged by confocal microscopy from the lower side of the leaf will appear darker when compared with those located at more abaxially positioned sites, since light scattering and absorption by cells that occur in the light path weaken excitation light intensity and reduce emissions. To address this limitation, calcofluor white fluorescence of general cell wall staining was used as an internal standard to calibrate AtSWEET11–GFP fluorescence intensities of different PP cells. In each minor vein, the relative fluorescence intensity of SWEET11–GFP in PP cells relative to PP TCs was calculated using the formula:


$$\begin{array}{l} F=\frac{S_{2}\times C_{1}}{S_{1}\times C_{2}} \end{array}$$

where F is the relative fluorescence intensity. Fluorescence intensity reads in each comparison were as follows: S_1_, SWEET11–GFP in PP TCs; S_2_, SWEET11–GFP in PP cells; C_1_, calcofluor white staining in PP TCs; and C_2_, calcofluor white staining in PP cells.

The relative fluorescence intensity of SWEET11–GFP in PP TCs was normalized to 1.

In a PP TC, the relative fluorescence intensity of SWEET11–GFP at sites with no wall ingrowths relative to sites containing wall ingrowth deposition was calculated using the formula described above. Fluorescence intensity reads in each comparison were: S_1_, SWEET11–GFP at the site with wall ingrowths; S_2_, SWEET11–GFP at the site with no wall ingrowths; C_1_, calcofluor white staining at the site with wall ingrowths; and C_2_, calcofluor white staining at the site with no wall ingrowths.

The relative fluorescence intensity of SWEET11–GFP at the site with wall ingrowths was normalized to1.

### Measurement of AtSWEET11 enrichment

The ratio of AtSWEET11 abundance comparing sites with and without wall ingrowth deposition was determined by measuring the accumulation of SWEET11–GFP fluorescence. Confocal images were processed in ImageJ as described above. For each PP TC, the fold change in AtSWEET11 abundance resulting from wall ingrowth deposition was calculated using the formula:


$$\begin{array}{l} FC=\frac{A_{1}\times L_{2}}{A_{2}\times L_{1}} \end{array}$$

where FC is the relative enrichment of plasma membrane area associated with the wall ingrowth relative to the side of the PP TC with no wall ingrowths. Reads of the accumulation area of AtSWEET11–GFP and primary cell wall length in ROIs were as follows: A_1_, the sum of SWEET11–GFP fluorescence at the site with wall ingrowths; A_2_: the sum of SWEET11–GFP at the site with no wall ingrowths; L_1_: primary cell wall length at the site with wall ingrowths; and L_2_: primary cell wall length at the site with no wall ingrowths.

Thus, this equation measures the relative fluorescence intensity per unit length of cell wall. These measurements were then compared with the wall ingrowth score in each corresponding PP TC.

## Results

### Basipetal progression of PP TC induction during development of Arabidopsis leaves

Previous studies indicated that PP TCs with extensive wall ingrowth deposition are distributed uniformly throughout juvenile leaves, whereas, in adult leaves, wall ingrowth deposition shows a distinctive basipetal gradient across the leaf, being more abundant in apical PP TCs (Nguyen *et al.*, 2017; Wei *et al.*, 2020). Two questions arise from this observation: how is the basipetal distribution pattern formed in adult leaves, and is the developmental progression of PP TCs in juvenile leaves different from that in adult leaves? To answer these questions, a time-course survey was conducted in developing leaves 1 and 7 in which the extent of PP TC development and wall ingrowth deposition in veins was scored using the method of Nguyen *et al.* (2017).

**Fig. 1.**
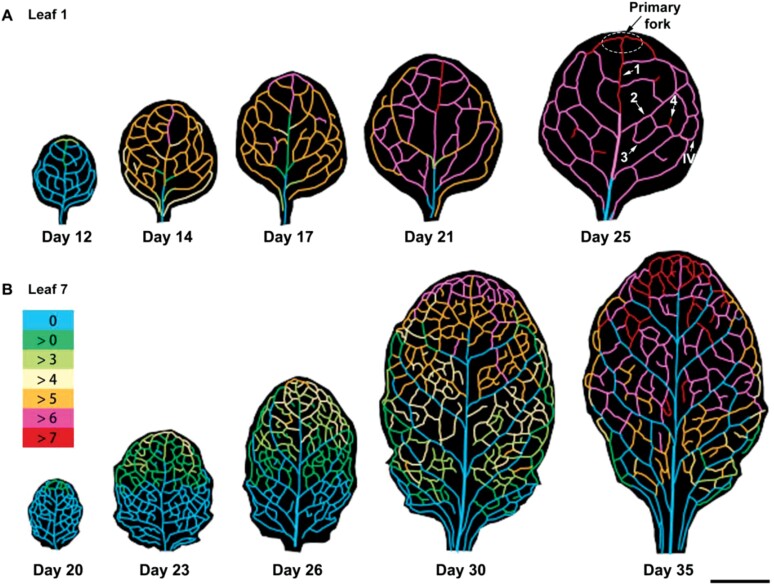
Schematic illustration of PP TC development in Arabidopsis leaves. Wall ingrowths in soil-grown Col-0 plants were quantified following Nguyen *et al.* (2017) in different sections of veins of each order. The shapes and vein patterns of the representative images are based on leaf scans, with the different colours representing wall ingrowth deposition scores derived from 3–7 leaves at each growth stage, and from 1–4 confocal images per vein. (A) Juvenile leaves (leaf 1) from 12- to 25-day-old plants. (B) Leaf 7 from 20- to 35-day-old plants. Vein classes are indicated by arrows in leaf 1 of a 25-day-old plant: (1) midrib, (2) secondary vein, (3) tertiary vein, (4) quaternary vein, and (IV) intramarginal vein. The primary fork is circled and indicated by an arrow. The key in (B) shows the colours denoting the PP TC score. Scale bar: 0.5 cm.

Based on the order of their formation and position, veins in Arabidopsis leaves can be classified into five orders (Kang *et al.*, 2007) as indicated in Fig. 1A. The onset of wall ingrowth deposition first occurred at the leaf apex and then gradually proceeded to the leaf base (Fig. 1). Consequently, the veins of the primary fork (the first vein that forms branching from the midrib, the central vein of the leaf) started to form ingrowths earlier than other veins, and thus deposited more abundant wall ingrowths in both leaves 1 and 7 (Fig. 1). In addition, in mature leaf 1, a basipetal gradient was observed in the midrib (Fig. 1A; Supplementary Fig. S1). Moreover, in secondary veins of developing leaf 1, the basal ends of secondary veins showed delayed formation of wall ingrowths when compared with the apical ends (Fig. 1A). Together, these results clarify the mechanism for the basipetal development of PP TCs in Arabidopsis adult leaves—wall ingrowth deposition occurs earlier in the leaf apex than in the leaf base rather than occurring simultaneously at these sites, and accumulates faster in the apical sites compared with the basal sites. These developments coincide with the functional maturation of minor veins in leaves (Wright *et al.*, 2003), and also indicate that the development of PP TCs in leaf 1 also follows a basipetal pattern, although not as dramatically as seen in leaf 7.

Generally, veins of the same order that were close to the leaf apex had higher levels of ingrowth deposition compared with veins located in the leaf base (Fig. 1). Nevertheless, in mature leaves, lower order veins (indicated by 3 and 4 in Fig. 1) formed later but displayed more abundant wall ingrowths compared with higher order veins (indicated by 1 and 2 in Fig. 1), suggesting that wall ingrowth deposition in PP TCs is coordinated with vein order. Notably, wall ingrowth deposition accumulated more rapidly in the juvenile foliar tissues (leaf 1) compared with leaf 7 which formed later during growth. Progression of wall ingrowth development from a score of <3 to >7 occurred across a 9 d window in leaf 1, but over 2 weeks in leaf 7. Together, these results suggest that the development of wall ingrowths in foliar tissues follows an underlying basipetal pattern but with temporal differences present between leaves of different developmental status.

### Distribution and morphology of PP TCs in minor veins of mature leaves

**Fig. 2.**
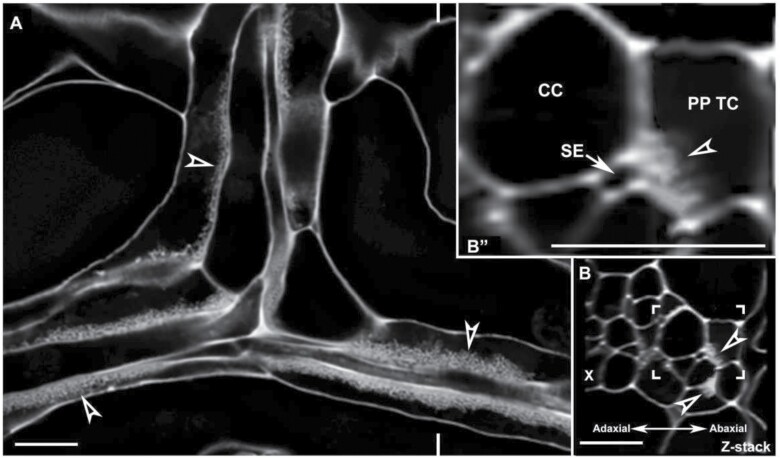
PP TC morphology and typical distribution in minor veins of mature leaves as assessed by confocal microscopy. (A) A single confocal section from an optical stack. In minor veins of a mature leaf, wall ingrowth deposition in PP TCs was abundant and highly polarized. Levels of wall ingrowth deposition varied in different PP TCs (arrowheads). (B) Orthogonal reconstruction of a confocal *Z*-stack through a vein shown in (A) at the location marked by indented lines indicated that most wall ingrowth deposition occurred at sites adjacent to the PP/SE interface. (Bʹʹ) Magnified image of the boxed region shown in (B). Note that wall ingrowths (arrowhead) in the PP TC occur primarily adjacent to the SE (arrow). Arrowheads indicate wall ingrowths; PP TC, phloem parenchyma transfer cell; SE, sieve element, indicated by an arrow; CC, companion cell; X, xylem. Pictures are representative images from different veins. Scale bars: 10 µm.

**Fig. 3.**
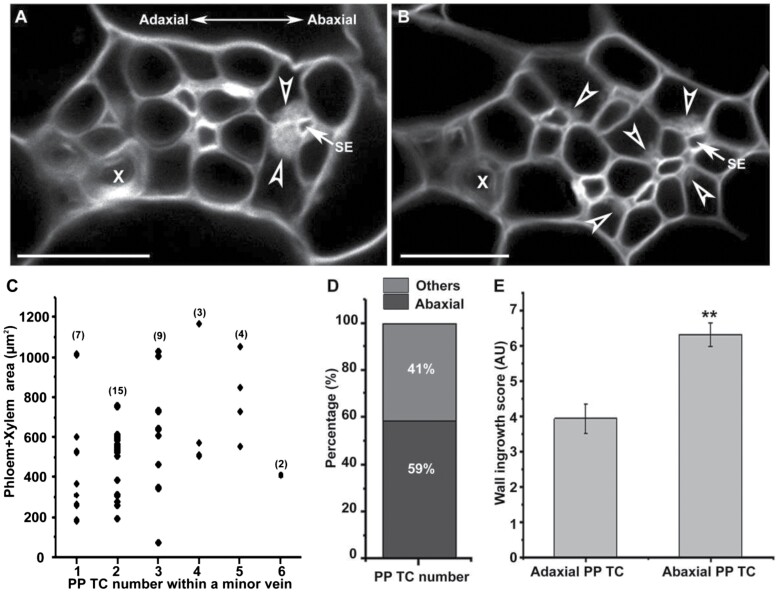
Wall ingrowth deposition was more abundant in abaxially positioned PP TCs than in adaxially positioned PP TCs. Confocal images were collected from mature leaf 7 from 5-week-old seedlings with inflorescences. (A) A typical leaf minor vein with two abaxially positioned PP TCs containing wall ingrowths (arrowheads). (B) A leaf minor vein with multiple PP TCs (indicated by arrowheads denoting wall ingrowth deposition) positioned at different sites throughout the phloem. (C) Counts of the number of PP TCs per size of vascular bundle (phloem+xylem area) derived from cross-sections of 40 minor veins. No correlation was seen between the number of PP TCs and the combined area of xylem and phloem cells, indicating that vein size does not control PP TC development. Numbers in parentheses indicate the replicates from each minor vein class. (D) Proportions of PP TCs located on different sites of the phloem in leaf minor veins. From 339 PP TCs present in 156 minor veins, 200 PP TCs were abaxially positioned PP TCs (defined as directly adjacent to or just one cell away from the abaxial bundle sheath cells) while 139 PP TCs were located in the middle and adaxial sites of the phloem. (E) Wall ingrowth scores were higher in abaxially compared with adaxially positioned PP TCs. Wall ingrowth scores were determined in eight minor veins (from three leaves) with multiple PP TCs, with the comparison made between the most adaxially and most abaxially positioned PP TC cells. Arrowheads indicate wall ingrowth deposition; PP TC, phloem parenchyma transfer cell; SE, sieve element (indicated by an arrow); X, xylem. Asterisks in (E) indicate significant differences (Student’s *t*-test, *P*<0.01). Samples for (A–D) were vibratome-cut cross-sections whereas (E) used whole mount leaves. Scale bars: 10 µm.

While wall ingrowth deposition in PP TCs has been studied by electron microscopy, the distribution of PP TCs relative to other phloem cells in individual veins remains unclear. Assessing the relative location of PP TCs in minor veins was investigated by collecting confocal optical stacks (Fig. 2A) and generating orthogonal reconstructions that were cross-sections (Fig. 2B). Typically, a mature minor vein possessed two rows of PP TCs which were both located on the abaxial side of the phloem within the vascular bundle (Figs 2B, 3A). However, a survey of 40 minor veins indicated that a relatively large percentage of minor veins had more than two PP TCs (~45% as shown in Fig. 3C), and that these veins contained not only paired PP TCs localized on the abaxial side of the phloem but also additional PP TCs that were scattered throughout the vascular bundle (Fig. 3B, C). Moreover, analysis of minor vein size, calculated as the combined area of phloem and xylem tissues, suggested that the number of PP TCs in a minor vein was not correlated with vein size (Fig. 3C). A survey on 156 individual minor veins was conducted to further clarify the relative location of PP TCs in minor veins. Counting the PP TCs that were directly adjacent to or just one cell away from the abaxial bundle sheath cells as the abaxial PP TCs, 59% of PP TCs located on the abaxial side of the phloem, while PP TCs on other sites (on the adaxial and middle sites) accounted for 41% (Fig. 3D). Moreover, abaxial PP TCs had the highest levels of wall ingrowth deposition compared with PP TCs positioned on the adaxial side of the phloem which had significantly lower levels (Fig. 3E; Supplementary Fig. S2). Together, these observations indicate that the distribution of PP TCs in the phloem is more concentrated in the abaxial half of the phloem, and wall ingrowth deposition showed a gradient, with abundance generally decreasing from the abaxial to the adaxial side of the phloem.

### Developmental and spatial progression of wall ingrowth deposition in leaf minor veins

**Fig. 4.**
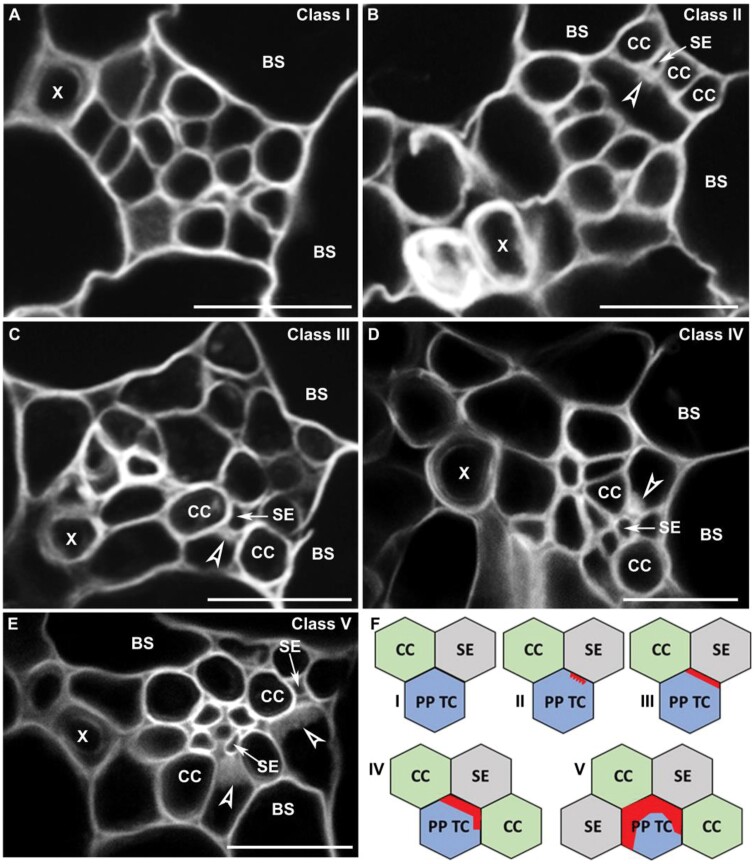
Development of wall ingrowth deposition in PP TCs in relation to neighbouring SEs and CCs. Cross-section images were collected from vibratome-cut cross-sections through maturing leaf 7 of 4- to 5-week-old Col-0 seedlings and the extent of wall ingrowth deposition in PP TCs was grouped into five representative classes. (A) Class I—no discernible wall ingrowths; (B) Class II—nascent wall ingrowth deposition positioned at the PP TC/SE interface; (C) Class III—substantial levels of wall ingrowth deposition entirely covering the PP TC/SE interface; (D) Class IV—extensive levels of wall ingrowth deposition cover the entire PP TC/SE interface and a small portion of the PP TC/CC interface; (E) Class V—massive levels of wall ingrowth deposition cover the entire PP TC/SE interface and considerable portions of neighbouring PP TC/CC interfaces. (F) Schematic figures illustrating the five representative classes of the extent of wall ingrowth deposition in PP TCs. Arrowheads indicate wall ingrowth deposition; arrows indicate sieve elements (SE); Red blocks in (F) indicate areas of wall ingrowth deposition as defined by Class I–Class V; PP TC, phloem parenchyma transfer cell; CC, companion cell; BS, bundle sheath; X, xylem. Images are representative images from three or more independent samples. Scale bars: 10 µm.

As shown in Fig. 3B, the first indications of wall ingrowth deposition in PP TCs of mature minor veins occurred almost exclusively at the interface with SEs, suggesting a possible association between the localized deposition of wall ingrowths in PP TCs and adjacent SEs. To further investigate this potential association, confocal images of cross-sections of maturing leaf 7 from 4- to 5-week-old seedlings were surveyed. Following the scoring system introduced by Nguyen *et al.* (2017), five categories of wall ingrowth deposition were defined. Class I PP cells showed no discernible wall ingrowths (Fig. 4A). In Class II, nascent wall ingrowths emerged at the PP TC/SE interface as evidenced by bristle-like structures along the uniform primary cell wall (Fig. 4B). In Class III, wall ingrowths in PP TCs were more obvious and covered the entire PP TC/SE interface and sometimes covered a small section of the adjacent PP TC/CC interface (Fig. 4C), while in Class IV, wall ingrowths were more abundant and not only covered the entire interface with SEs but also covered a considerable proportion of the adjacent interface with CCs (Fig. 4D). In Class V, wall ingrowths reached the highest level of deposition, with some PP TC/CC interfaces being entirely covered (Fig. 4E). These observations indicated that wall ingrowths were first deposited adjacent to SEs but then gradually extended to cover adjacent PP TC/CC interfaces, as indicated in the schematic diagram (Fig. 4F).

### Vascular structure and relative localization of wall ingrowths in mature minor veins in Arabidopsis leaves

TCs develop wall ingrowths to facilitate enhanced rates of transmembrane transport of solutes (Pate and Gunning, 1972). In mature PP TCs, wall ingrowths cover the interface adjacent to the SE/CC complex (Haritatos *et al.*, 2000; Amiard *et al.*, 2007; Nguyen *et al.*, 2017), suggesting the existence of solute transport at the joint interface. However, the precise relationships between wall ingrowths in the PP TCs relative to SEs and CCs are poorly understood. Consequently, the function of PP TCs and their potential interactions with other phloem cells have not been fully elucidated. Thus, to further understand the positioning of wall ingrowths relative to other cell types, in particular CCs, we used the transgenic line *pAtSUC2::AtSTP9-GFP* (*tmSTP9*). The AtSTP9–GFP fusion expressed in this line is anchored to the CC plasma membrane and is a non-mobile CC marker (Stadler *et al.*, 2005). By staining cross-sections of minor veins from this line with calcofluor white to reveal wall ingrowths, it was possible to unambiguously identify CCs and PP TCs to enable the quantification of these cell types within vascular bundles.

**Fig. 5.**
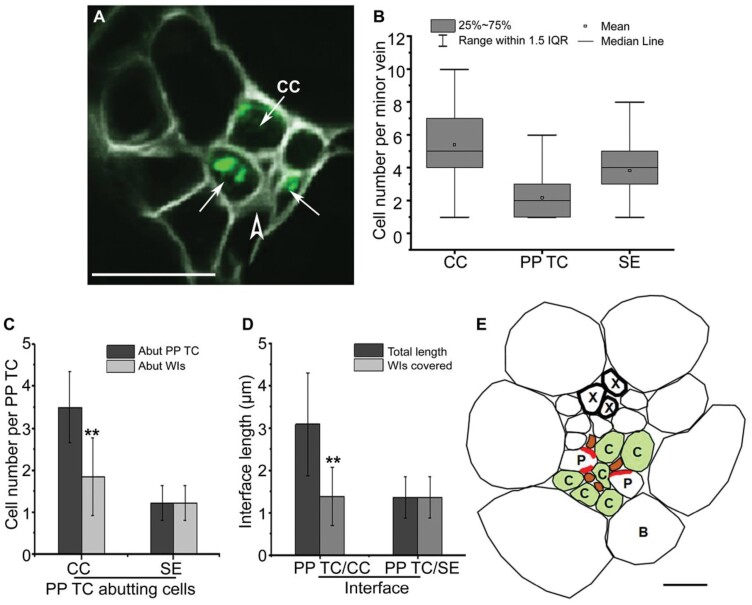
Minor vein structure and localization of wall ingrowth deposition in leaf vascular tissue in Arabidopsis. (A) Confocal imaging of a vibratome cross-section through an fixed minor vein from mature transition leaf 7 of the *pAtSUC2:AtSTP9-GFP* line expressing AtSTP9–GFP under control of the *pAtSUC2* promoter and localized to the plasma membrane of CCs (arrows). Sections were counter-stained with calcofluor white. Arrows indicate AtSTP9–GFP-expressing CCs, with the plasma membrane plasmolysed as a consequence of fixation. The arrowhead indicates wall ingrowths in the PP TC. (B) Phloem cell numbers in each minor vein of mature leaves. The average cell numbers were obtained from cross-sections of 145 leaf minor veins. (C) Numbers of CCs and SEs that abut a PP TC (*n*=36 PP TCs from 26 independent minor veins) (dark grey) and numbers of CCs and SEs around a PP TC that abut wall ingrowths (light grey). (D) Lengths of PP TC/CC (*n*=160) and PP TC/SE (*n*=84) interfaces, and lengths of the PP TC/CC and the PP TC/SE interfaces containing wall ingrowths. (E) Schematic diagram of vascular components in a minor vein of mature Arabidopsis leaves. X, xylem; P, PP TC; C, CC; B, bundle sheath. Red blocks in the PP TCs in (E) represent wall ingrowths. Asterisks in (C) and (D) indicate a significant difference by the Student’s *t*-test (**P<0.01). Scale bars: 10 µm.

Consistent with previous observations made by TEM (Haritatos *et al.*, 2000; Amiard *et al.*, 2007), our fluorescence observations confirm that wall ingrowth deposition in PP TCs in mature leaves occurs only at the interface of the cell wall adjacent to SEs and CCs (Fig. 5A), supporting the role of PP TCs in phloem loading (Chen, 2014). However, as shown in Fig. 5A, not all CCs in minor veins lie adjacent to PP TCs, suggesting that the phloem loading path in leaf minor veins could be complex. From observations of 145 leaf minor veins, and counting cell types in minor veins in mature leaves 6, 7, and 8, the average numbers of CCs, SEs, and PP TCs are about five, four, and two, respectively, in each minor vein (Fig. 5B).

The relative proportions and locations of the different cell types were also quantified. On average, one PP TC abuts ~3.5 CCs and 1.2 SEs, but only 55% of the CCs lie adjacent to wall ingrowths (Fig. 5C, E). In contrast, SEs that abut PP TCs all lie adjacent to wall ingrowths (Fig. 5C, E). Furthermore, for those PP TC/CC interfaces in which wall ingrowths are present, only 50% of the interfaces were covered by wall ingrowths, whereas entire interfaces with SEs were covered with wall ingrowths (Fig. 5D, E). Interestingly, as shown in Fig. 5D, the average length of wall ingrowths along PP TC/CC interfaces was almost equal to that of the PP TC/SE interface, being 1.39 µm and 1.36 µm, respectively. Together, these observations indicate that the association between wall ingrowths in PP TCs and abutting SEs is tighter than that with the abutting CCs in leaf minor veins in Arabidopsis.

### Development of PP cells and PP TCs in leaf minor veins in Arabidopsis

AtSWEET11 is a sucrose transporter localized to the plasma membrane of PP TCs where it functions to export sucrose from PP TCs into the apoplasm adjacent to the SE/CC complex in leaf minor veins in Arabidopsis (Chen *et al.*, 2012; Cayla *et al.*, 2019). However, a recent study using scRNA-seq analysis demonstrated that leaf PP cells expressing *AtSWEET11* can be classified into two distinct clusters. Genes involved in callose deposition and cell wall thickening are over-represented in one PP cluster (PP1), whereas genes involved in photosynthesis are enriched in the second cluster (PP2) (Kim *et al.*, 2021). These data imply the existence of two distinct populations of PP cells in Arabidopsis leaves.

**Fig. 6.**
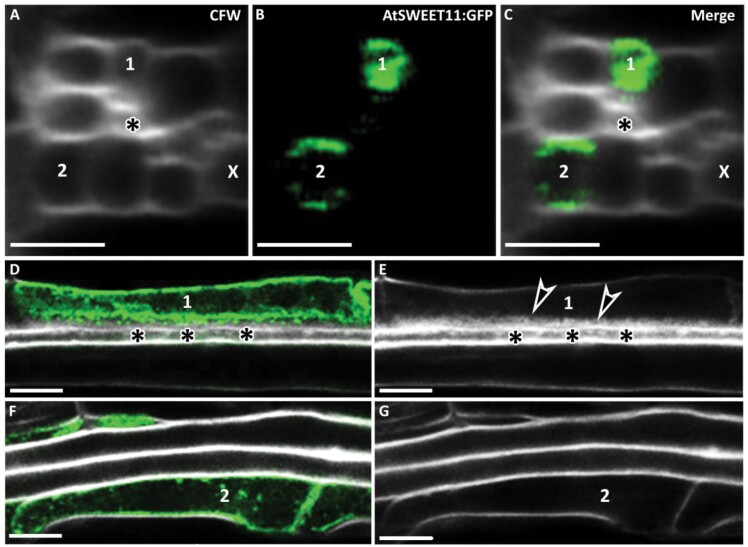
Adjacent SEs are required for wall ingrowth deposition in PP TCs in minor veins of Arabidopsis. Leaf 1 from 27-day-old seedlings of the transgenic line *pAtSWEET11::AtSWEET11-GFP* was fixed and cleared using ClearSee, stained with calcofluor white (CFW), and imaged by confocal microscopy. (A–C) Cross-sections were generated from confocal optical stacks using orthogonal sectioning. (A) Cross-section of a minor vein in a mature juvenile leaf stained with CFW and showing two PP cells (labelled 1 and 2) and an adjacent SE (asterisk). (B) AtSWEET11–GFP fluorescence in PP cells 1 and 2. (C) Merged image of (A) and (B), indicating the presence of two PP cells showing AtSWEET11–GFP expression. PP cell 1 was adjacent to an SE (asterisk) and showed strong AtSWEET11–GFP fluorescence, whereas PP cell 2 showed weaker fluorescence and was not immediately adjacent to an SE. (D, E) PP cell 1 expressing AtSWEET11–GFP (D) showed abundant wall ingrowth deposition (arrowheads in E) on the interface adjacent to an SE (asterisk). (F, G) PP cell 2 had no detectable wall ingrowth deposition (G), but expressed AtSWEET11–GFP (F). X, xylem. Arrowheads indicate wall ingrowth deposition. Scale bars: 5 µm.

Based on these observations, the hypothesis arises that PP TCs with wall ingrowths belong to the PP1 cluster, while PP cells with no wall ingrowths belong to the PP2 cluster. Previous studies have suggested that PP cells expressing *AtSWEET11* and *AtSWEET12* have wall ingrowths (Chen *et al.*, 2012; Cayla *et al.*, 2019). However, whether all PP cells in leaf minor veins have wall ingrowths, thus defining them as PP TCs, remains unknown. To clarify this question, mature juvenile leaf 1 from 27-day-old seedlings of the *pAtSWEET11::AtSWEET11:GFP* transgenic line was observed by confocal microscopy. However, PP TCs are located in vascular bundles, which makes imaging of PP TCs in whole leaves particularly challenging. Therefore, peeling away the abaxial epidermis as described by Cayla *et al.* (2019), followed by fixation and ClearSee treatments (Kurihara *et al.*, 2015), were applied. Cleared samples were then stained with calcofluor white to observe cell walls. In Fig. 6, a minor vein containing two cell files expressing AtSWEET11–GFP, thus identified as PP cells, is presented. Orthogonal reconstructions showed two PP cells, labelled 1 and 2 (Fig. 6A–C) with different characteristics. For example, PP cell 1 was adjacent to an SE (asterisk) but PP cell 2 was not. More importantly, PP cell 1 had transdifferentiated into a PP TC, as evidenced by abundant wall ingrowth deposition along its interface adjacent to the SE (Fig. 6D, E). In contrast, PP cell 2 had no detectable levels of wall ingrowth deposition (Fig. 6F, G), indicating that not all AtSWEET11–GFP-expressing PP cells in the Arabidopsis leaf minor vein had transdifferentiated to become PP TCs. However, not all observed minor veins showed this pattern, with analysis of 20 minor veins from eight different leaves revealing that only five minor veins contained PP cells expressing AtSWEET11–GFP but not depositing wall ingrowths to become PP TCs (Fig. 6F; Supplementary Fig. S3C, D). In most cases, however, multiple PP TCs within a minor vein all deposited wall ingrowths along the interface adjacent to the SE, as shown in Supplementary Fig. S3A, B. Together, these observations suggest that minor veins have two subtypes of PP cells, those not adjacent to SEs and having no discernible wall ingrowth deposition, and the others being adjacent to SEs and having transdifferentiated to become PP TCs as a consequence of wall ingrowth deposition. Hence, these results demonstrate the tight association of wall ingrowth deposition in PP TCs with neighbouring SEs.

### Accumulation of AtSWEET11–GFP in PP cells and PP TCs

**Fig. 7.**
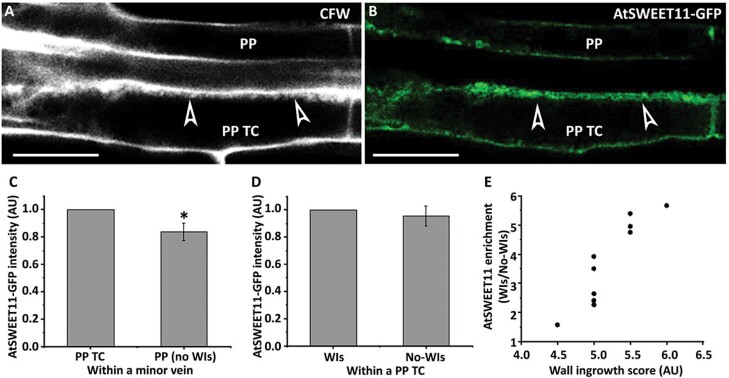
PP TCs have higher levels of AtSWEET11 compared with PP cells. Leaf 1 from 27-day-old seedlings was fixed in 4% (w/v) formaldehyde, cleared using ClearSee solution, and stained with calcofluor white. (A) Cell wall labelling using calcofluor white. Arrowheads indicate wall ingrowth deposition in the PP TC, but no deposition in the PP cell. (B) AtSWEET11–GFP fluorescence of the same region shown in (A). (C) Relative fluorescence intensity of AtSWEET11–GFP in PP TCs and PP cells in minor veins. (D) Relative fluorescence intensities of AtSWEET11–GFP in PP TCs where wall ingrowth (WI) deposition had occurred or not. Four pairs of PP TCs and PP cells from four different leaf samples were compared and analysed to generate (C) and (D). (E) AtSWEET11 enrichment, as measured by the ratio of AtSWEET11–GFP fluorescence on the side of the cell with wall ingrowths against the opposite side of the cell, as a result of an enlarged plasma membrane area caused by wall ingrowth deposition. Data were obtained from 10 PP TCs in eight different leaves. The asterisk in (C) indicates a significant difference by Student’s *t*-test (*P<0.05). Pictures are representative images from four independent samples. Scale bars: 10 µm.

Since wall ingrowth deposition in TCs results in increased plasma membrane surface area and AtSWEET11 is localized to the plasma membrane in PP TCs, it is reasonable to assume that AtSWEET11–GFP should be more abundant in PP TCs that have wall ingrowths compared with PP cells that have no wall ingrowths. To investigate this possibility, fluorescence intensity was quantified in foliar minor veins of *pAtSWEET11::AtSWEET11-GFP* leaves that had been peeled, fixed, and processed with ClearSee. In minor veins, the AtSWEET11–GFP signal was stronger in PP TCs compared with PP cells in which no wall ingrowth deposition had occurred (Fig. 7A–C). This analysis supports the qualitative observations in Fig. 6 and Supplementary Fig. S3. Statistical analysis indicated that the fluorescence intensity of AtSWEET11–GFP in PP cells was ~84% of the signal intensity detected in PP TCs (Fig. 7C). However, fluorescence intensity of the AtSWEET11–GFP signal showed no distinct difference between sites with wall ingrowths and those without (Fig. 7D), indicating that AtSWEET11 is relatively evenly distributed along the plasma membrane in PP TCs. Nevertheless, as shown in Fig. 7E, the increase in plasma membrane surface area caused by ingrowth deposition resulted in localized AtSWEET11 enrichment. For instance, compared with sites with no wall ingrowths, AtSWEET11 abundance at sites with a wall ingrowth score of five was ~3-fold higher than sites with no wall ingrowths (Fig. 7E). Therefore, consistent with a role in phloem loading, PP TCs possess higher levels of AtSWEET11 compared with PP cells with no wall ingrowths, which is achieved not only by the enlarged plasma membrane surface area but also by the higher localized density of AtSWEET11.

### Wall ingrowth deposition is not affected in the *suc2-1* mutant

**Fig. 8.**
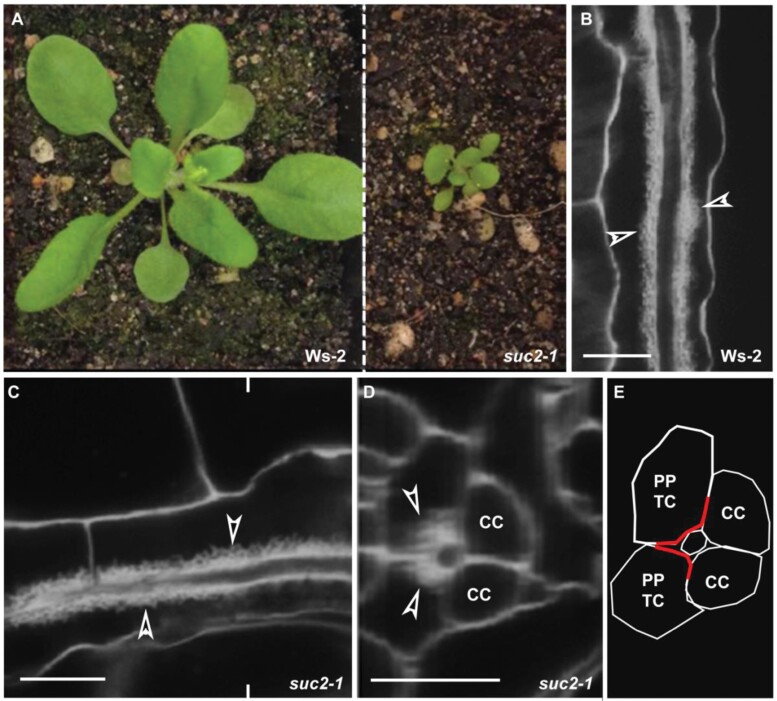
Wall ingrowth deposition is unaffected in *suc2-1* plants. Confocal images were collected from leaf 1 of 25-day-old Ws-2 (wild-type control) and heterozygous *suc2-1*^*+/–*^ plants. In 25-day-old Ws-2 plants, PP TCs were fully mature in leaf 1 (Nguyen *et al.*, 2017). (A) *suc2-1* seedlings were much smaller than Ws-2. (B, C) Wall ingrowth deposition was abundant in leaf minor veins of both Ws-2 (B) and *suc2-1* (C). (D) Orthogonal reconstruction of a confocal *Z*-stack through the *suc2-1* vein shown in (C) at the location marked by indented lines. (E) Schematic image of phloem cells shown in (D). Arrowheads in (B–D) indicate wall ingrowth deposition in PP TCs. Red lines in (E) represent interfaces covered by wall ingrowths. Scale bars: 10 µm.

We sought to investigate the control by the SE/CC complex on the localization of wall ingrowth deposition through the use of mutants. Not surprisingly for such fundamental processes as phloem development and transport, comparatively few mutant lines are available. The *suc2-1* mutant, deficient in the sucrose transporter that loads the phloem, is homozygous lethal, and the growth of even the *suc2-1*^*+/–*^ heterozygous mutant is highly compromised (Gottwald *et al.*, 2000) (Fig. 8A). However, wall ingrowths formed in leaf 1 of 25-day-old *suc2-1*^*+/–*^ plants were similar to those observed in the Ws-2 control (Fig. 8B, C), and reconstructions demonstrated that wall ingrowths in mature leaves of the mutant covered a large proportion of the PP TC/CC interface (Fig. 8D, E).

## Discussion

### Onset of wall ingrowth deposition in PP TCs is coupled with functional maturation in Arabidopsis leaf minor veins

The results presented in this study illustrate the spatial and temporal developmental progression of PP TCs in Arabidopsis. The onset of PP TC development in veins proceeds basipetally and starts at a relatively later stage of leaf development when vascular patterning is close to completion (Fig. 1). Consequently, at day 12 in leaf 1 and day 20 in leaf 7, vein structure was nearly complete but wall ingrowths had only just started to form (Fig. 1). Thus, wall ingrowths do not form until the vein is structurally mature. In addition, the order of PP TC development does not match the order in which vascular patterning proceeds across leaves. For instance, the first loop of secondary veins develops acropetally and outwards from the midrib (Scarpella *et al*., 2006, 2010). PP TC development, however, starts from the apex site of the loop, indicated as the primary fork in Fig. 1A. Moreover, subsequent vein loops form in a bidirectional manner (Scarpella *et al*., 2006, 2010), with development initiated from both the previous loop and the midrib, whereas PP TC development in these vein loops always started from the section attached to the previous loop, particularly in leaf 7 (Fig. 1). These observations indicate that the formation of wall ingrowths in PP TCs is not coupled with the structural maturation of the vein. This conclusion is further supported by the observation that some higher order minor veins, such as quaternary veins that are formed last (Kang *et al.*, 2007), will develop similar or even more extensive levels of wall ingrowth compared with the lower order, secondary and tertiary veins that are formed earlier in leaf development (Fig. 1). In addition, wall ingrowth deposition does not occur in shaded regions of the leaf where the minor veins are structurally mature (Wei *et al.*, 2020). The lack of coordination between formation of ingrowths and structural maturation of the vein is also consistent with the observation that even when the leaf is mature, high light will stimulate additional ingrowth deposition (Amiard *et al.*, 2005).

Coinciding with the development of wall ingrowths, the sink to source transition (functional maturation process) in foliar tissues also proceeds from the tip to the base (Wright *et al.*, 2003; Wei *et al.*, 2020). Moreover, consistent with the observation in a previous study (Nguyen *et al.*, 2017), the heteroblastic development of PP TCs was observed with distinct differences present between leaf 1 and leaf 7 (Fig. 1). Compared with leaf 7, the basipetal gradient of the sink to source transition was much less obvious in leaf 1 in Arabidopsis (Supplementary Fig. S4), which is consistent with their temporary difference in wall ingrowth deposition. Together, these observations showed a tight correlation between wall ingrowth deposition and the functional maturation process of the phloem, which not only provides further evidence for the positive correlation between wall ingrowth deposition and phloem loading activity in PP TCs, but also offers new insights into the underlying mechanism for the heteroblastic development of PP TCs in Arabidopsis.

### Arabidopsis leaf minor veins contain two subtypes of PP cells

Several recent studies have demonstrated the structural and functional complexity of the phloem tissue in plants. In grasses such as maize and rice, two types of SEs have been identified, one thin walled and symplasmically connected to CCs but no other cell type, and the other thick walled with abundant plasmodesmatal connections to vascular parenchyma cells (McCubbin and Braun, 2021). Furthermore, in maize leaves, abaxially positioned bundle sheath cells specifically express three SWEET sucrose transporters (SWEET13a, b, and c) which are all involved in phloem loading, while adaxial bundle sheath cells do not express these transporters, indicating at least two different bundle sheath populations in maize leaves (Bezrutczyk *et al.*, 2021). In Arabidopsis and tobacco leaf minor veins, two types of CCs have also been identified, one expressing *FT* and the other not (Chen *et al.*, 2018). A recent single-cell RNA expression study also demonstrated the complexity of phloem, with Kim *et al.* (2021) reporting that PP cells can be transcriptionally divided into two subclusters, a PP1 cluster enriched in cell wall genes and genes involved in transmembrane transport, and a PP2 cluster enriched in photosynthesis genes (Kim *et al.*, 2021).

These observations demonstrate that phloem structure in leaves is complex and that phloem cell population including SEs, CCs, and bundle sheath cells are functionally diverse. The results reported in this study expand this complexity to include PP cells in Arabidopsis minor veins and suggest the presence of two subtypes of PP cells—one subtype adjacent to SEs and containing wall ingrowths, thus defining them as PP TCs, and the other subtype of PP cells in the same minor vein which do not develop wall ingrowths and were not adjacent to SEs (Fig. 6). Compared with PP cells, PP TCs not only contained wall ingrowths but also more strongly expressed *AtSWEET11* (Figs 6, 7), indicating morphological and physiological differences between these two subtypes of PP cells. Together, these results strongly suggest the presence of two subtypes of PP cells in leaf minor veins in Arabidopsis. In this context, it would be interesting to investigate whether the PP1 cluster identified by Kim *et al.* (2021) corresponds to PP TCs and thus the PP2 cluster would correspond to PP cells.

### Phloem loading might preferentially occur via abaxial PP TCs in Arabidopsis leaves

Results from this and a previous study (Wei *et al.*, 2020) demonstrate that wall ingrowth deposition is positively correlated with phloem loading activity in PP TCs. Therefore, wall ingrowth deposition could be regarded as a trait, similar to root growth, which indicates phloem loading activity in Arabidopsis. In most mature minor veins, PP TCs mostly develop on the abaxial side of the phloem (Figs 2B, 4B–E, 5A). Moreover, PP TCs are more active in phloem loading as they possess higher levels of AtSWEET11 compared with non-transdifferentiated PP cells that have no wall ingrowths (Fig. 7). Furthermore, in minor veins that have multiple PP TCs, wall ingrowth deposition levels also show a distinct gradient so that PP TCs on the abaxial side of the phloem accumulated more abundant wall ingrowths in comparison with the PP TCs on the adaxial side (Fig. 5). Thus, one might hypothesize that the phloem loading activity in PP TCs that are localized on the abaxial side of the phloem might be higher than that of those on the adaxial side. Evidence from scRNA-seq analysis of maize leaves established that sucrose uptake into phloem is mainly via abaxial bundle sheath cells (Bezrutczyk *et al.*, 2021). Consistent with this observation, the path of sucrose export in maize minor veins is through the abaxially positioned thin-walled SEs (reviewed by McCubbin and Braun, 2021). These observations demonstrate that phloem loading activity is asymmetric in maize minor veins, which supports the hypothesis developed here regarding the different functions for abaxial and adaxial PP TCs in phloem loading in Arabidopsis.

### Wall ingrowth deposition in PP TCs is tightly associated with adjacent SEs in leaf minor veins in Arabidopsis

A body of evidence, including observations in this study, indicates that wall ingrowth deposition in PP TCs in Arabidopsis polarizes to the interface adjacent to the SE/CC complex (Haritatos *et al.*, 2000; Amiard *et al.*, 2007; Adams *et al.*, 2014). As shown in Fig. 4, in Arabidopsis minor veins, wall ingrowths first occurred at the PP TC/SE interface and then gradually extended across the adjacent PP TC/CC interface, implying a potential impact of SEs on the initiation of wall ingrowths in PP TCs. Moreover, all the PP TC/SE interfaces were totally covered by wall ingrowth deposition, while less than half of the total length of the PP TC/CC interfaces were covered similarly (Fig. 5). This result complicates the assumption that wall ingrowths in PP TCs are developed by the plant mainly to facilitate solute exchange between PP TCs and CCs. Furthermore, none of the minor vein cross-section images collected in this study showed any PP TCs with wall ingrowths that were not adjacent to SEs. However, observations in the *pAtSWEET11:AtSWEET11:GFP* transgenic plants indicated that a population of PP cells did not form discernible wall ingrowths, and these PP cells were not adjacent to the SEs (Fig. 6; Supplementary Fig. S3C, D). Together, the data reported here indicate that the transdifferentiation of PP cells into PP TCs is tightly associated with adjacent SEs in Arabidopsis.

### Wall ingrowth deposition in PP TCs is not coordinated with the phloem loading activity of CCs

In classical phloem loading models, neither symplasmic nor apoplasmic loading pathways have evoked PP TC/SE interactions in the collection veins. Instead, the typical loading route proposes that sucrose exported from PP cells (or PP TCs) is actively taken up into CCs via SUC2/SUT1 and then delivered symplasmically into SEs for long-distance transport (Turgeon and Wolf, 2009; Schepper *et al.*, 2013; Chen, 2014). Thus, the polarized distribution of early wall ingrowth deposition, predominantly correlating spatially with SEs rather than CCs, seems peculiar. However, our previous study indicated that wall ingrowth deposition is not coordinated with expression levels of the CC-specific sucrose transporter gene *SUC2* (Wei *et al.*, 2020). In addition, although growth of the *suc2-1*^*+/–*^ heterozygous mutant is highly compromised (Gottwald *et al.*, 2000), wall ingrowths were abundant in mature leaf 1 from 25-day-old mutant plants and similar to wild-type controls (Fig. 8). Moreover, wall ingrowths in PP TCs still covered a large portion of the PP TC/CC interface in *suc2-1* plants, indicating that the impaired loading activity of CCs in the mutant had little effect on wall ingrowth deposition in neighbouring PP TCs. In addition, TEM images from a study by Amiard *et al.* (2007) show that wall ingrowths in PP TCs only occur at the regions adjacent to SEs regardless of whether or not they are adjacent to CCs. In contrast, PP TC/CC interfaces that are not adjacent to SEs have no wall ingrowth deposition, which is consistent with observations in this study. In conclusion, the observations reported here and in previous studies suggest that wall ingrowth deposition in PP TCs is not coordinated with phloem loading activity in CCs.

### Reconsidering the role of wall ingrowth deposition in phloem loading in Arabidopsis leaves

Wall ingrowth deposition in PP TCs promotes phloem loading by enlarging the plasma membrane surface area of the exporting PP TCs. However, our previous study showed that wall ingrowth formation is induced by phloem loading, rather than wall ingrowth deposition itself being a prerequisite for phloem loading (Wei *et al.*, 2020). Super-resolution imaging of wall ingrowths has demonstrated that these structures can increase the available plasma membrane surface area for transporters involved in phloem loading (Rae *et al.* 2020); however, until these ingrowths extensively cover the PP TC wall, their contribution to phloem loading will only be minimal. In addition, mature transition and adult leaves show low levels of wall ingrowth deposition towards the base of the leaf, with scores of <4 (Nguyen *et al.*, 2017; Fig. 1) which seems to preclude a role for wall ingrowths in facilitating transmembrane transport of photo-assimilates in these cells. Furthermore, as discussed in this study, wall ingrowth deposition preferentially covers the PP TC/SE interface rather than the CC/SE interface (Figs 3–5). However, the PP/CC interface is proposed to be the site where phloem loading of sucrose occurs (Chen, 2014). Together, these observations indicate that wall ingrowth deposition in PP TCs may have additional roles rather than solely functioning in facilitating sucrose transport. Interestingly, the steady-state pattern of hydrostatic pressure distribution in the leaf phloem network, which is largely determined by leaf shape, coincides with the heteroblastic pattern of wall ingrowth deposition observed in Arabidopsis (reviewed by Wei *et al.*, 2021). The hydrostatic pressure in the leaf is caused by solute uptake in collection phloem (Gould *et al.*, 2005). Similar to the distribution of wall ingrowth deposition in Arabidopsis leaves, the distribution of hydrostatic pressure in narrower/longer leaves shows a basipetal gradient, whereas it is relatively uniform in wider/shorter leaves (Sakurai and Miklavcic, 2021). These similarities imply the existence of a possible association between wall ingrowth deposition and water transport in Arabidopsis leaves.

### Conclusions

We have demonstrated that wall ingrowth deposition enhances phloem loading activity by increased accumulation of the sucrose transporter AtSWEET11 in PP TCs. Moreover, results from this study refine the temporal and spatial development of PP TCs, which provides further evidence for the impact of phloem loading activity on wall ingrowth deposition in PP TCs (Wei *et al.*, 2020). The asymmetric distribution patterns of wall ingrowth deposition across minor veins in Arabidopsis, in turn, imply that phloem loading activity in Arabidopsis leaves is heteroblastic, and might be mainly undertaken by abaxial PP TCs in minor veins. However, our observations demonstrate that the influence of SEs on PP TC development is more dominant compared with that of CCs, implying potential interaction between PP TCs and SEs in phloem loading. Furthermore, results from this study also suggests that two subtypes of PP cells might exist in leaf minor veins in Arabidopsis that are distinguished by the presence of wall ingrowths and the abundance of AtSWEET11.

## Supplementary data

The following supplementary data are available at *JXB* online.

Fig. S1. PP TC morphology and distribution in the midrib of mature leaves.

Fig. S2. Representative images of adaxially and abaxially positioned PP TCs in a mature leaf minor vein.

Fig. S3. Representative images of mature minor veins in Arabidopsis leaves.

Fig. S4. Functional maturation status in maturing leaves in Arabidopsis.

erac234_suppl_supplementary_figures_S1-S4

## Data Availability

All data supporting the findings of this study are available within the paper and within its supplementary data published online.
