# Supplementary material for: Asymmetric wall ingrowth deposition in Arabidopsis phloem parenchyma transfer cells is tightly associated with sieve elements
Source: J Exp Bot. 2022 May 24;73(16):5414–27. doi: 10.1093/jxb/erac234 (PMC9467654; doi:10.1093/jxb/erac234)
Supplement: erac234_suppl_supplementary_figures_S1-S4 [file erac234_suppl_supplementary_figures_s1-s4.pdf]

Supplementary data

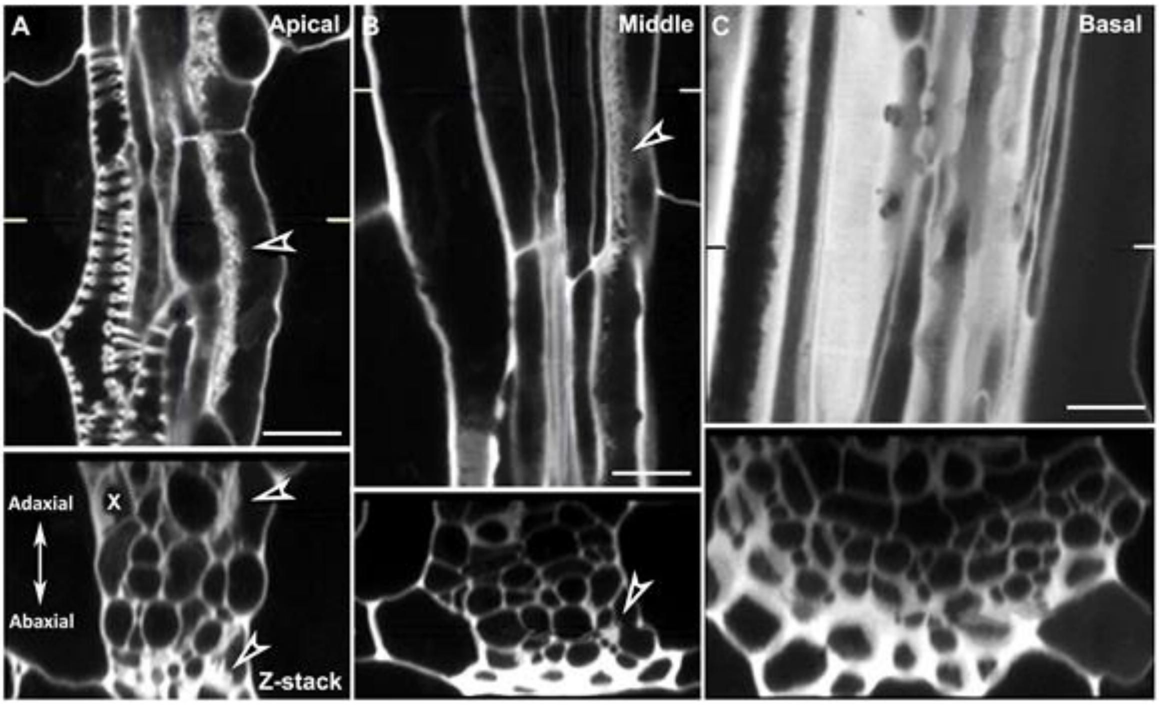

Supplemental Figure S1. PP TC morphology and distribution in the midrib of mature leaves. Confocal optical sections of propidium iodide-stained samples through the main vein at different locations in mature juvenile leaf 1, with the lower panels showing orthogonal reconstructions through the midrib at locations indicated by indent lines. (**A,** Apical) Wall ingrowth deposition (arrowhead) formed a gradient in the midrib, with the apical section showing the most abundant deposition of wall ingrowths. (**B,** Middle) Middle section of the mid-vein showing developed PP TCs (arrowhead) at the outer edge of the vascular tissue as well as thick-walled cells. (**C**, Basal) Basal sections were characterised by the absence of PP TCs and extensive development of thick-walled collenchyma cells. Scale bars: 10 µm.

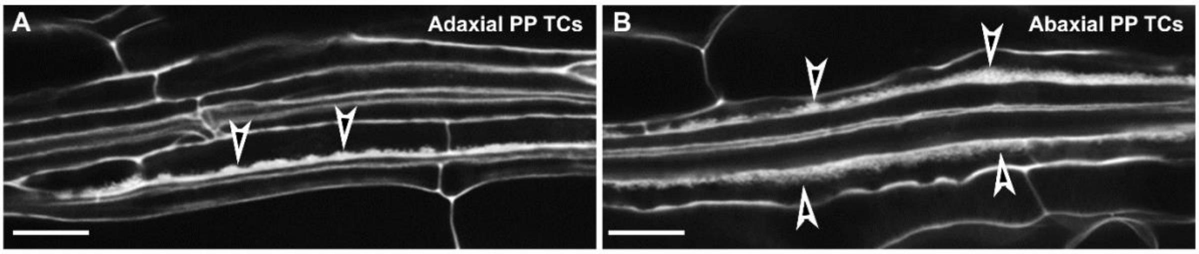

Supplemental Figure S2. Representative images of adaxially- and abaxially-positioned PP TCs in a mature leaf minor vein. Confocal images were collected from mature leaf 7 from 5-week-old seedlings with inflorescences. The extent of wall ingrowth deposition was much higher in abaxial PP TCs compared to adaxial PP TCs. (**A**) Adaxially positioned PP TC. (**B**) Abaxially positioned PP TC. Arrowheads indicate wall ingrowth deposition. Scale bars: 10 µm.

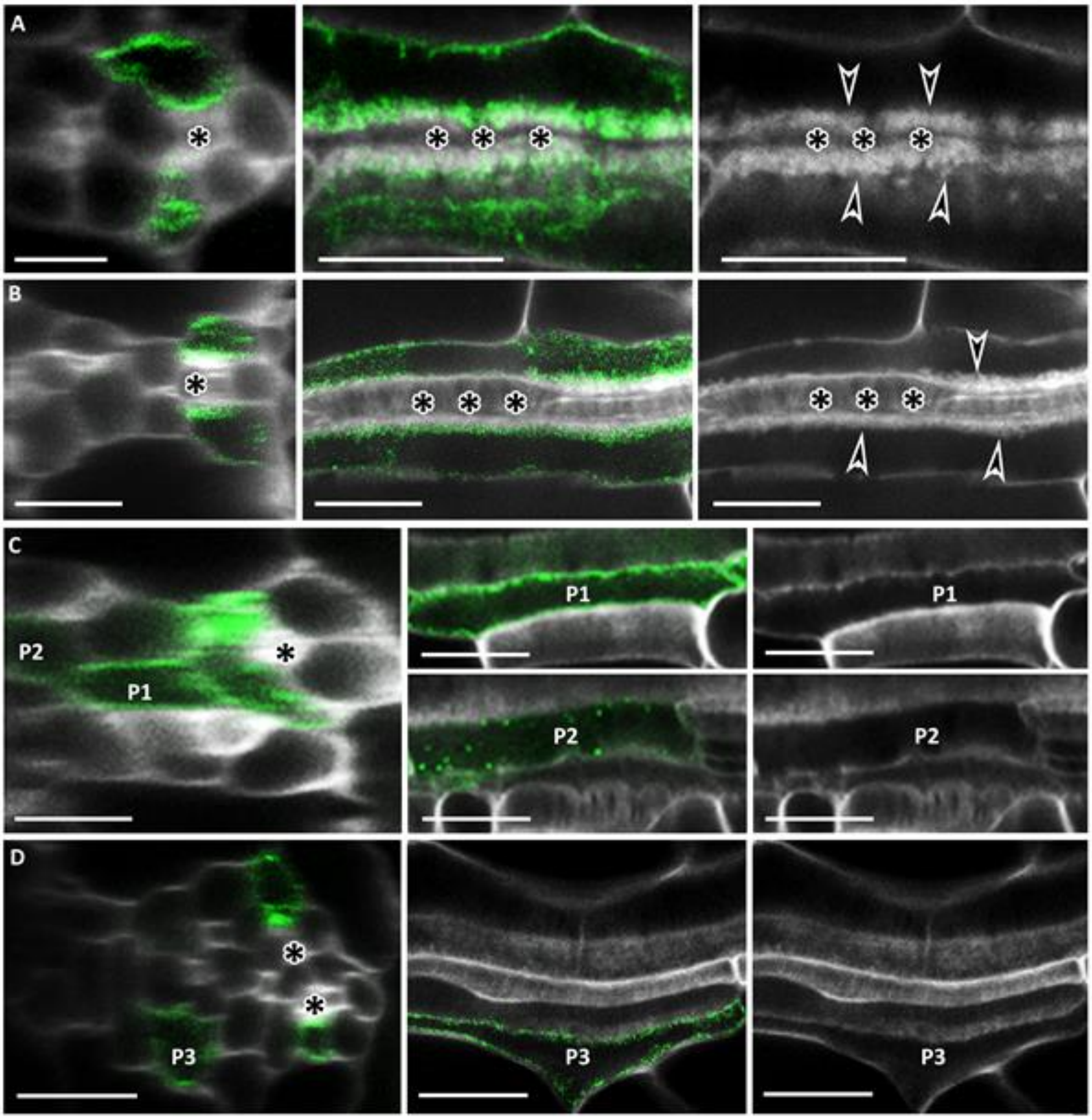

Supplemental Figure S3. Representative images of mature minor veins in Arabidopsis leaves. Images were collected from mature leaves of *pSWEET11::AtSWEET11:GFP*. Green signal represents AtSWEET11-GFP. Each image set has one orthogonal reconstruction image and two corresponding optical images. (**A**) and (**B**) Minor veins showing multiple PP cells that have become PP TCs via deposition of wall ingrowths. (**C**) and (**D**) Minor veins with PP cells. P1, P2, and P3 represent PP cells that did not develop wall ingrowths. Arrowheads indicate wall ingrowth deposition. Asterisks indicate SE. Scale bars: 10 µm.

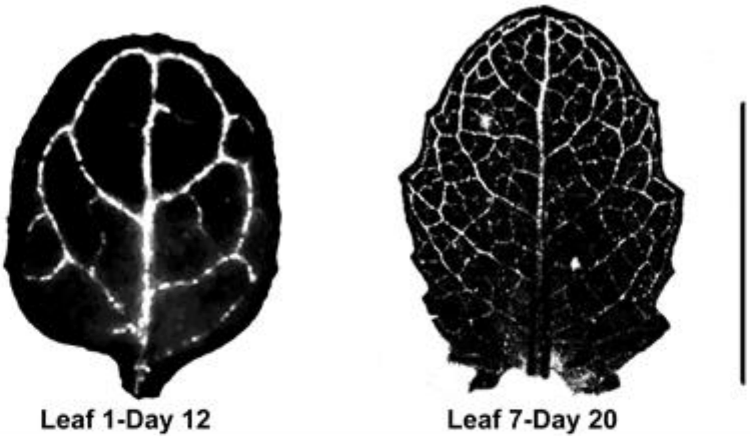

Supplemental Figure S4. Functional maturation status in maturing leaves in Arabidopsis. Images were taken on leaf 1 of a 12-day-old seedling, and leaf 7 from a 20-day-old seedling. Functional maturation status of the phloem is indicated by the presence of *pAtSUC2::AtSTP9-GFP*. In leaf 7, functional maturation status of the phloem showed a distinct basipetal gradient whereas this gradient was much less obvious in leaf 1. Scale bar: 5 mm.
